# Insufficient natural killer cell responses against retroviruses: how to improve NK cell killing of retrovirus-infected cells

**DOI:** 10.1186/s12977-016-0311-8

**Published:** 2016-11-08

**Authors:** Elisabeth Littwitz-Salomon, Ulf Dittmer, Kathrin Sutter

**Affiliations:** Institute for Virology, University Hospital in Essen, University of Duisburg-Essen, Hufelandstr. 55, 45147 Essen, Germany

## Abstract

Natural killer (NK) cells belong to the innate immune system and protect against cancers and a variety of viruses including retroviruses by killing transformed or infected cells. They express activating and inhibitory receptors on their cell surface and often become activated after recognizing virus-infected cells. They have diverse antiviral effector functions like the release of cytotoxic granules, cytokine production and antibody dependent cellular cytotoxicity. The importance of NK cell activity in retroviral infections became evident due to the discovery of several viral strategies to escape recognition and elimination by NK cells. Mutational sequence polymorphisms as well as modulation of surface receptors and their ligands are mechanisms of the human immunodeficiency virus-1 to evade NK cell-mediated immune pressure. In Friend retrovirus infected mice the virus can manipulate molecular or cellular immune factors that in turn suppress the NK cell response. In this model NK cells lack cytokines for optimal activation and can be functionally suppressed by regulatory T cells. However, these inhibitory pathways can be overcome therapeutically to achieve full activation of NK cell responses and ultimately control dissemination of retroviral infection. One effective approach is to modulate the crosstalk between NK cells and dendritic cells, which produce NK cell-stimulating cytokines like type I interferons (IFN), IL-12, IL-15, and IL-18 upon retrovirus sensing or infection. Therapeutic administration of IFNα directly increases NK cell killing of retrovirus-infected cells. In addition, IL-2/anti-IL-2 complexes that direct IL-2 to NK cells have been shown to significantly improve control of retroviral infection by NK cells in vivo. In this review, we describe novel approaches to improve NK cell effector functions in retroviral infections. Immunotherapies that target NK cells of patients suffering from viral infections might be a promising treatment option for the future.

## Background

Despite more than 30 years of intensive research, HIV still represents a global health problem with up to 37 million HIV-infected people worldwide in 2015. After infection with HIV, the human immune system is not able to fully control the virus, which finally results in the development of the lethal acquired immunodeficiency syndrome (AIDS). HIV preferentially infects human leucocytes like macrophages and T cells carrying the surface protein CD4 and the co-receptor CXCR4 or CCR5. The progression to AIDS is accompanied with a decline in CD4^+^ T cell numbers. However, the reasons for the failure of the host immune system in HIV infection are complex. To date, there is no cure or vaccine available, but antiretroviral therapy (ART) can control the progression of the disease for decades.

To develop new strategies to combat retroviral infections, animal models are required to analyze host immune responses against retroviruses and their modulation by various immunotherapies. As mice cannot be infected with HIV-1, murine retroviruses should be used to discover basic concepts of innate and adaptive immunity in retroviral infections. The mouse model that has been used most intensively to study retroviral immunity in the past is the infection of mice with the Friend Retrovirus (FV) complex. The FV complex, consisting of the Friend murine leukemia virus (F-MuLV) and the Spleen focus-forming virus (SFFV), induces acute splenomegaly in susceptible mice due to a rapid polyclonal erythroblast proliferation and transformation, which is subsequently followed by the development of a lethal erythroleukemia [1]. However, resistant mouse strains mount a potent antiviral immune response during the acute phase of infection that can prevent the onset of leukemia [2]. Despite this initial viral control, FV eventually escapes from T cell mediated immunity and establishes a chronic infection [3]. This mouse model does not resemble pathological features of HIV-1 infection, but there are many similarities in innate and adaptive immune responses during HIV-1 and FV infection [4]. The development of chronic infection is associated with dysfunctionality of effector CD8^+^ T cells and the activation and expansion of regulatory T cells in HIV-1 and FV infection [5, 6]. NK cell responses were also shown to control acute infections with FV or HIV-1 [7, 8]. Thus, the FV model allows to study NK cell functions during acute retroviral infection in great detail and to therapeutically manipulate the NK cell response in retroviral infections in vivo.

## NK cell biology

The first line of immune responses against viruses is mediated through innate immune cells. As a part of the innate immune system NK cells are responsible for early antiviral functions. NK cells express various germline-encoded inhibitory and activating receptors, like natural cytotoxicity receptors (NCRs), C-type lectin-like receptors and killer cell immunoglobulin-like receptors (KIRs) in humans and the corresponding Ly49 receptors in mice. A dysbalance of signals from these receptors can lead to activation of NK cells. NK cell effector functions include cytotoxicity and production of cytokines and chemokines. In humans, NK cells represent 2–18% of the lymphocytes in human peripheral blood [9] and are comprised of two main subsets, characterized by bright CD56 (CD56^bright^) or low-density CD56 (CD56^dim^) expression [10]. CD56^dim^ NK cells constitute more than 90% of the NK cells in the peripheral blood and they are able to induce apoptosis of virus-infected cells by the release of granzymes and perforin or binding of ligands (TRAIL, FasL) to their death receptors (TRAIL-R, FasR). The majority of this NK cell subset expresses CD16 (Fcγ receptor III) [10], which is necessary for ADCC and critical for NK cell mediated lysis of HIV-infected cells [11]. A minority of NK cells are CD56^bright^ (10%), a subset that efficiently produces a variety of cytokines such as interferon-γ (IFN-γ), tumor necrosis factor-α (TNF-α), granulocyte–macrophage colony-stimulating factor (GM-CSF) and IL-10 upon activation [12]. In mice, NK cells can be characterized with the C-type lectin NK1.1 and CD49b, which in combination defines mature NK cells. Equivalent to CD56 in human NK cell subsets, murine NK cells expressing CD27 are primarily cytokine producers whereas CD27^−^ CD11b^+^ NK cells exhibit a cytolytic phenotype [13, 14]. Maturation and activation of NK cells correlates with DC activity and cytokine production whereas NK cells are also able to alter DC functions, reviewed in detail elsewhere [15, 16]. Crosstalk between NK cells and DCs is often followed by maturation of DCs and an upregulation of NK cell effector functions. Production of type I IFN by plasmacytoid DCs (pDCs) or release of IL-12, IL-15 and IL-18 by conventional DCs as well as direct cell–cell contact result in higher cytokine production and improved cytotoxicity of NK cells [17–21]. Cytokines such as IFN-γ and TNF-α produced by NK cells mediate maturation and increase functionality of DCs [22]. On the other hand, NK cells can eliminate immature DCs via recognition by the activating receptor NKp30 [19]. Additionally, production of immunosuppressive IL-10 by NK cells further dampens not only DC activation but also pleiotropic immune responses [23].

## HIV therapy: today and future prospects

Currently, more than 37 million people are infected with HIV. Despite great progress in HIV research, there is still no cure from HIV infection. Treatment with antiretroviral drugs can diminish viremia below detection limit, but eradication of HIV from viral reservoirs or activation of the immune system to control HIV infection is still a task for the future. Moreover, there are numerous disadvantages of ART. ART requires life-long treatment with high costs, drug resistance development as well as side effects of the medication. There are different therapeutic approaches for new HIV therapies to overcome these problems: (1) Eradication cure with the objective to completely eliminate HIV from all compartments of the body. (2) Hybrid cure aiming at reducing viral reservoirs as well as improving virus control without the usage of ART. (3) Functional cure with the intention to control HIV replication without using ART [24]. Several of these approaches to induce HIV immune control are currently under investigation, like broadly neutralizing antibody therapies, usage of recombinant viral vectors to achieve induction of cytotoxic CD8^+^ T cells as well as genome editing or expansion and activation of virus-specific immune cells. Modification of NK cell activity should also be considered since NK cells can directly control retroviral infections and regulate virus-specific T cell responses [25].

## Sensing of retroviruses and the impact on NK cell responses

**Fig. 1:**
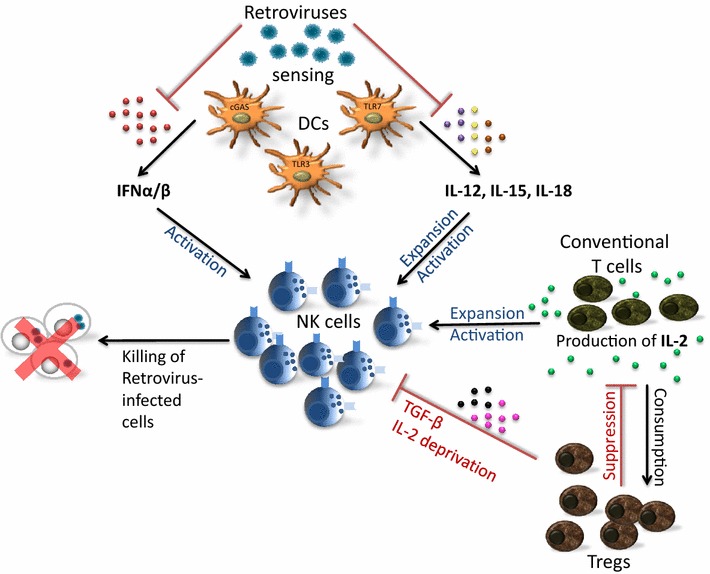
NK cell influencing factors. cGAS, TLR7 and TLR3 are important for the sensing of retroviruses. The sensing results in the production of cytokines that stimulate NK cell responses. NK cells represent an important immune cell subset, which contributes to the control of retroviral infections. Retroviruses can actively suppress molecular or cellular factors that are required for NK cell activation. Immunosuppressive cytokines such as TGF-β or cytokine deprivation by Tregs also suppress NK cell effector functions. NK cell activity can be enhanced by therapeutic stimulation with exogenous cytokines (IL-2, IL-12, IL-15, IL-18) and by IFNα/β. *cGAS* cyclic GMP-AMP synthase, *TLR* toll-like receptors, *IL* interleukin, *IFN* interferon, *TGF* transforming growth factor

Sensing of viruses is utilized by a variety of different host pattern recognition receptors (PRRs). During the replication cycle of retroviruses like HIV, simian immunodeficiency virus (SIV) or MuLV, various nucleic acid intermediates are generated (reviewed in [26]), which might be potential targets for endosomal or cytoplasmic sensors. Up to now, it is not completely understood, which PRRs are essential for the recognition of HIV or murine retroviruses and how this sensing further influences host immune responses. During HIV infection TLR7/8 senses GU-rich ssRNA in pDCs and monocytes leading to an antiviral state in the infected and bystander cells by the induction of type I IFN (Fig. 1) [27, 28]. Other important sensors for retroviruses located in the cytosol are RIG-I [29], cGAS [30, 31], and the IFNγ-inducible protein 16 (IFI16) [32, 33]. During murine retrovirus infections TLR3 and TLR7 are required for efficient viral sensing and induction of innate and adaptive immune responses [34–37]. During Friend Retrovirus (FV) infection of mice deficiency in TLR3 resulted in decreased IFNα expression and impaired NK and CD8^+^ T cell cytotoxicity [37]. Immune sensing of viral infections can also induce inflammasome activation [38]. Inflammasomes are cytosolic, multimeric protein complexes that integrate several endogenous and exogenous signals. Formed inflammasomes regulate caspase-1, which proteolytically activates IL-1β and IL-18 [39], which further influences NK cell responses. It has been reported that inflammasome activation is induced during HIV infection in monocytes and macrophages [38], which may in turn influence NK cell responses.

## NK cell responses in retroviral infections

Healthy cells are protected from NK cell-mediated killing primarily by the expression of Major histocompatibility complex I or Human Leukocyte antigen (HLA)-C and HLA-E. Their interaction of these molecules with inhibitory receptors on NK cells suppresses the NK cell response. Decreased expression of HLA-A and HLA-B on HIV-1 infected target cells has been described as immune evasion strategy to avoid recognition by cytotoxic CD8^+^ T cells, but this escape from adaptive immunity can render HIV-1 infected cells susceptible for NK cell killing [40]. NK cells can eliminate retrovirus infected target cells in vitro and in vivo through direct lysis or via ADCC [7, 8, 41]. Especially in acute retroviral infections (HIV-1 in humans, SIV in macaques, and FV in mice) elevated NK cell numbers and augmented cytolytic activity of NK cells could be detected [7, 42–44]. Moreover, depletion of NK cells during early FV infection showed an increase in virus infected cells in mice [7]. Activated NK cells can also produce chemokines that bind to the CCR5 receptor (MIP-1α = CCL3, MIP-1β = CCL4, RANTES = CCL5) and block the entry of R5-tropic HIV strains into CD4^+^ target cells by competitive prevention of receptor binding [45, 46]. This inhibitory effect on HIV replication through chemokine release is impaired in the presence of high HIV viremia [47]. The secretion of the antiviral cytokines IFN-γ and TNF-α by NK cells can also mediate suppressive effects on retrovirus replication and does not correlate with the level of plasma viremia [47, 48]. Whereas NK cells clearly play an antiviral role in acute retroviral infections, they may have different effects during the chronic infection phase. [7]. During chronic FV infection or persistent infections like LCMV, NK cells negatively regulate virus-specific CD4^+^ and CD8^+^ T cell responses, influencing adaptive immunity and possibly contributing to viral chronicity (reviewed in [49–51]). Furthermore, during other chronic viral infections, such as HIV and hepatitis C virus (HCV), a third CD56^negative^ NK cell population arises (up to 15–38% of the total NK cells), which represents a dysfunctional population with lytic deficiencies and secretory dysregulations [42, 52, 53].

## Counter-regulation of the NK cell response by retroviruses

### Viral proteins and peptides

The importance of the NK cell response in retroviral infections became obvious when researchers discovered that HIV-1 actively counter-regulates their response. Interactions of activating receptors such as NKG2D and its ligand retinoic acid early transcript-1 (RAE-1) are essential for the activation of NK cells. In FV-infected mice, interactions of NKG2D–RAE-1 were important for NK cell-mediated killing of infected target cells [54]. Thus, retroviruses developed mechanisms to downregulate ligands for activating receptors on infected cells to escape from NK cell killing. For example, ligands for the NK cell receptors NKG2D (CD314), DNAM-1 (CD226) and NKp44 (CD336) are counter-regulated by the HIV-1 proteins Nef (negative regulatory factor) and Vpu (viral protein U), an inhibitory mechanism to prevent activation of NK cells [55–58]. In plasma of HIV-1 patients the release of soluble ligands for NKG2D detuned signaling of this activating receptor and resulted in its downregulation [57].

During the last years, several combinations of NK cell receptors (KIRs) and their ligands (HLA alleles) were identified, which are beneficial or detrimental for the outcome of HIV infection and disease progression (reviewed elsewhere [59]). Interestingly, activation or inhibition of NK cells also depends on the recognition of peptides bound to HLA molecules. Recent data highlight the importance of HIV-1-derived peptides presented on HLA molecules and the impact of small mutations within these peptides for binding of NK cell receptors [60]. Especially the carboxyl terminus and in particular the residues at positions 7 and 8 seem to be of specific relevance for the initiation and the strength of binding [61]. Sequence polymorphisms within regions of HIV-1 that are targeted by inhibitory KIRs improved the binding and resulted in decreased antiviral activity of NK cells [62, 63]. In contrast, HIV sequence mutations can also prevent binding of inhibitory receptors to HLA molecules, thereby increasing the susceptibility of infected cells to lysis [64].

### Immunological dysregulation of NK cell responses during retroviral infections

Beside NK cell evasion mechanisms that are directly mediated by HIV-1 proteins or peptides, retrovirus infections can also dysregulate molecules or cells of the immune system to circumvent NK cell activation. One example is that retroviruses counter-regulate type I IFN responses, which are in turn critical for NK cell activation and differentiation (Fig. 1). It has been reported that IFNα is only transiently induced during acute human and simian retroviral infections (HIV, SIV). Plasmacytoid DCs are the main producers of type I IFNs, but they become infected by HIV due to the expression of CD4 on their surface. As a consequence the blood pDC levels decline during HIV infection, which reduces the IFN response [65, 66]. In addition, HIV downregulates IRF3 especially in CD4^+^ T cells and thus suppresses the induction of type I IFN [67, 68]. During chronic HIV infection IFN-α might be associated with disease progression. It has been suggested that persistent IFN-α production by pDCs during chronic HIV infection contribute to hyper immune activation [69–71]. Blockade of IFNα receptor prevented the activation of HIV-exposed CD4^+^ and CD8^+^ T cells [72]. Additionally, treatment of SIV-infected macaques with human pegylated IFN-α2a led to a decline of induced ISGs after repeated IFN application resulting in an IFN desensitized state [73]. Treated animals expressed elevated levels of FOXO3a, a negative regulator of IFN signaling, and increased depletion of CD4^+^ T cells was observed, although no changes in viral loads were detectable. During the infection of mice with murine retroviruses type I IFN is undetectable in the serum [74, 75] and Lin et al. [76] recently showed, that Moloney Leukemia Virus (MLV)-based vectors directly block the production of type I IFN in pDCs. The molecular mechanism of this suppression remains currently unknown. Our own studies with FV-infected mice have demonstrated that mice lacking the receptor for type I IFN have viral loads comparable to wild type mice [77], suggesting that the type I IFN response is significantly reduced by the virus. Nevertheless, exogenous application of type I IFN subtypes during acute FV infection can activate NK cells and reduce viral replication and infection-induced disease [78, 79].

During many infections myeloid DCs secrete IL-12 or IL-15 that potently induce NK cell activation and proliferation (Fig. 1). In turn, NK cells secrete cytokines like IFN-γ, TNF-α and GM-CSF, which lead to the maturation of myeloid DCs and further increase their cytokine production. During HIV infection, infected DCs can be found in the blood of untreated patients [80–82], however replication of the virus is very inefficient in DCs compared to activated CD4^+^ T cells [83, 84] due to the expression of different host restriction factors in DCs. Many studies showed that HIV-exposed or infected DCs have an altered cytokine expression profile [85–87]. In particular, the secretion of IL-12 by myeloid DCs is strongly reduced in HIV-infected individuals resulting in decreased proliferation of NK cells and diminished IFN-γ secretion by NK cells [88, 89]. Similar results were also described in acutely SIV infected macaques [90]. In vitro infection of DCs with HIV also led to a defective production of IL-12 and IL-18 [91]. In contrast to IL-12 and IL-18, increased IL-15 levels were detected in the serum and lymph nodes of untreated HIV-infected individuals [92–95], which especially activates CD8^+^ T cells, but they did not analyze NK cells.

Several studies have shown that regulatory T cells (Tregs) can suppress homeostatic NK cell responses [96, 97]. We and others reported that acute retroviral infections (FV, HIV, SIV) expand and activate Tregs, which then suppress virus-specific T cell responses [5, 98, 99]. It was therefore likely that retrovirus-induced Tregs also inhibit antiviral NK cell responses. Indeed, we recently demonstrated that specific ablation of Tregs in FV infected mice improved the activation, cytokine production, and cytotoxic activity of NK cells [100]. Competition for IL-2 was found to be the main molecular mechanism of Treg–NK cell suppression (Fig. 1). The expanded Tregs consumed large amounts of IL-2 with their high-affinity trimeric IL-2 receptor resulting in deprivation of IL-2 from NK cells, which only express the low-affinity dimeric IL-2 receptor [101, 102]. Also other suppressive mechanisms, such as the expression of TGF-β (Transforming Growth Factor-β) by Tregs, have been described for the inhibition of NK cell activity [103, 104].

## Therapeutic approaches to augment NK cell responses in retroviral infections

### IFNα therapy

Endogenous expression of type I IFNs strongly influences NK cell responses during viral infections. Type I IFNs promote NK cell activation and cytotoxicity [105–112]. IFNα also regulates the expression of IFN-γ by NK cells in a Signal Transducers and Activators of Transcription (STAT) 4-dependent manner [113, 114]. Treatment of patients chronically infected with HCV showed that exogenous IFNα can polarize NK cells to a cytotoxic phenotype [115]. During acute FV infection of mice, application of IFNα (the IFNα1 and IFNα11 subtypes) improved NK cell responses resulting in reduced viral loads [78, 79]. This was a direct effect of IFNα on NK cells as shown by experiments with bone marrow chimeric mice [79]. Treatment of HIV-infected humanized mice with different subtypes of IFNα revealed a potent role of the subtype IFNα-14 in reducing viremia and proviral loads [116]. Furthermore, IFNα-14 therapy augmented the frequency of TRAIL^+^ NK cells whereas other subtypes did not alter the NK cell response. In vivo application of IFNα also restored perforin expression of NK cells in HIV-infected patients [117]. Similar effects were observed in HIV in vitro models. NK cell lysis of HIV-infected cells was strongly enhanced by CpG treatment and this effect was mediated by type I IFN [118].

The molecular mechanisms that are involved in IFN-induced NK cell activation are still not fully understood. The expression of type I IFN induces transcription of hundreds of IFN-stimulated genes (ISGs) with direct antiviral as well as immunomodulatory properties. Some of these expressed ISGs were shown to be important in controlling retroviral replications. These host restriction factors include apolipoprotein B mRNA-editing enzyme, catalytic polypeptide-like 3G (APOBEC3G, A3G), Tetherin, SAM and HD domain-containing protein 1 (SAMHD1), tripartite motif-containing protein 5α (TRIM5α), Myxovirus resistance 2 (MX2), Schlafen 11 (SLFN11) and IFN-induced transmembrane proteins (IFITMs) [119, 120]. Tetherin was shown to inhibit the release of retroviruses from infected cells by tethering nascent virions to the plasma membrane [121]. Despite this direct antiviral function, IFN-induced Tetherin also improved NK and T cell responses during acute FV infection [122]. Tetherin expression mediated increased surface expression of MHC class II and the costimulatory molecule CD80, as well as the production of IL-15 by DCs, which correlated with increased IFN-γ production and higher cytotoxicity of NK cells [123]. The authors hypothesized that the tethered virions may promote viral sensing by TLR3 or TLR7 upon endocytosis resulting in higher cytokine expression by DCs, which modulates NK cell functions. Various in vitro studies demonstrated that cells infected with HIV-1Δvpu cannot antagonize Tetherin. Thus, HIV-1Δvpu-infected cells have enhanced numbers of nascent virions tethered to the cell membrane and are indeed more susceptible to NK cell-mediated killing via ADCC [124–126]. The restriction factor A3G was also described to influence NK cell responses during retroviral infections. A3G belongs to the family of cytidine deaminases and is incorporated into progeny viruses in the absence of the HIV-1 viral infectivity factor (Vif). Upon new infections of other cells incorporated A3G deaminates cytidine to uridine during reverse transcription, resulting in hypermutations in the provirus and degradation of newly synthesized DNA strands [127, 128]. A3G can also directly inhibit reverse transcription [129]. Norman et al. demonstrated that the HIV-1 viral protein (Vpr) binds to uracil DNA glycosylase 2 (UNG2), which was activated through A3G-mediated deamination processes. This interaction induced DNA-damage repair mechanism results in higher surface expression of NKG2D ligands in infected cells and subsequently improved NK cell cytotoxicity [130]. Thus, therapeutic application of IFNα directly activates NK cells, but can also augment the expression of host restriction factors, which further augment NK cell effector functions.

### Stimulation of NK cells with IL-2

IL-2 was discovered in the 1970s and was used for the first immunotherapy proved to be beneficial in patients with end-stage metastatic melanoma or renal cell carcinoma [131–134]. IL-2 was originally described as T lymphocyte stimulatory factor influencing important functions in survival, activation, proliferation and differentiation of various lymphocyte populations including NK cells [135–137]. The importance of this cytokine for NK cell activation and expansion was shown in experiments with IL-2 knockout mice [138, 139]. Interestingly, HIV elite controllers have significantly higher cytokine levels than progressors, especially for IL-2, IFN-γ and TNF-α [140, 141]. NK cells in elite controllers or long-term nonprogressors reveal higher activation and increased cytotoxic activity [142, 143]. In clinical trials with HIV progressors, treatment with IL-2 plus antiretroviral therapy increased the CD4^+^ T cell count but resulted in no additional clinical benefit [144]. Long-term treatment of HIV patients with intermittent IL-2 therapy mainly expanded CD25^+^ CD4^+^ regulatory T cells whereas it did not alter CD25 or CD122 expression on NK cells [145].

One reason for the up to now unsuccessful IL-2 therapy in HIV patients might be that different receptors for IL-2 (IL-2R) exist on distinct immune cell populations. There is a trimeric, high-affinity IL-2R and a dimeric, low-affinity receptor [146]. The high-affinity IL-2R consists of the subunits IL-2Rα (CD25), IL-2Rβ (CD122) and the common γ-chain (CD132) whereas the low-affinity receptor comprises of CD122 and CD132. Memory CD8^+^ T cells as well as NK cells express high levels of the low-affinity dimeric IL-2R on the cell surface, however, Tregs, activated CD4^+^ and CD8^+^ T cells predominantly express the high-affinity IL-2R [137]. Thus, standard IL-2 therapy mainly affects T cells, but not NK cell responses.

IL-2/anti-IL-2 monoclonal antibody complexes can overcome this problem. IL-2 in complex with anti-mouse IL-2 mAb S4B6 or anti-human IL-2 MAB602 is preferentially directed to the CD122 receptor subunit. Treatment with specific IL-2/anti-IL-2 mAb complexes also circumvents severe side effects of high dose IL-2 therapy and results in increased half-life of IL-2 in vivo [147, 148]. The IL-2 mAb S4B6 complex has already been shown to improve NK cell responses and subsequent clearance of tumor cells [100, 149]. In the FV model, an up to 90% reduction in viral loads was demonstrated after specific stimulation of NK cells with IL-2 mAb S4B6 complex [100]. In this study, proliferation and maturation of NK cells as well as activation and effector functions were significantly improved. The IL-2 mAb S4B6 complex therapy prohibited the consumption of the IL-2 by Tregs and made it available for NK cell stimulation. The study shows that targeted IL-2 therapy may be a new approach to selectively stimulate the antiviral activity of NK cells in retroviral infections.

### Enhancement of NK cell functions by IL-12, IL-15 or IL-18

Stimulation of NK cells with IL-2 antibody complexes is very potent for the reduction of tumor burden and viral replication but for retroviral infections complete viral control was not achieved by IL-2 stimulation so far. Combination therapy with other cytokines further augmenting NK cell functions, e.g. IL-12, IL-15 and IL-18, would be a good amendment. Interestingly, IL-15 shares numerous biologic properties with IL-2 such as the IL-2R/IL15R β-chain (CD122) and the common γ-chain (CD132), however IL-15 binds with high-affinity to its unique IL15Rα subunit (CD215). IL-15 is indispensable for the maturation of NK cells and IL-15Rα knockout mice completely lack NK cells [150, 151]. Similar to the biologic effect of IL-2, IL-15 efficiently activates NK cells and CD8^+^ T cells while it negligibly stimulates Tregs [101].

During acute HIV-1 infection, Stacey et al. [152] detected a correlation between an increase in plasma viremia and elevated IL-15 and IFNα levels. IL-15 levels seem to correlate with viral loads, since IL-15 therapy resulted in accelerated disease progression and augmented viral set points probably due to enhanced CD4^+^ target cell proliferation [153]. Furthermore, others have shown that IL-15 levels during acute SIV infection were associated with higher susceptibility of memory CD4^+^ T cells for SIV infection [154].

However, also promising results of IL-15 therapy in retroviral infections were obtained. Stimulation of NK cells with a superagonistic IL-15 antibody increased their cytotoxic activity and was able to inhibit acute HIV-1 infection in humanized mice [155]. Injections of recombinant IL-15 increased numbers of NK cells and effector memory CD8^+^ T cells in SIV infection, but surprisingly, no changes in viral set points were detected [156]. In melanoma models it was demonstrated that stimulation of effector cells with IL-15/IL-15Rα complexes or the IL-15 fusion protein RLI (composed of the N-terminal domain of IL-15Rα coupled via a linker to IL-15) significantly reduced tumor burden [157]. The increased elimination of melanoma cells post treatment with RLI was NK cell dependent [157, 158]. However, such IL-15 complex therapies have so far not been tested in retroviral infection models. Thus, the role of IL-15 in NK cell activation and especially its relevance for the treatment of retroviral infections has to be further investigated in future studies.

In 1991, IL-12 was first termed “natural killer cell stimulatory factor” due to its capacity to augment NK cell cytotoxicity, but it also increases the IFN-γ production and the lymphocyte proliferation [159]. The heterodimeric IL-12 consists of p35 and p40 subunits, which are shared by members of the IL-12 family like IL-23 and IL-35 [160].

In HIV-infected patients, the production of heterodimeric IL-12 is about fivefold reduced in comparison to healthy controls [161, 162]. Thus, HIV patients may benefit from therapy with exogenous IL-12.

Treatment with the biologic active IL-12 (p70) induced high IFN-γ levels and protected mice from murine acquired immunodeficiency syndrome (MAIDS) [163]. Furthermore, reduced viral loads and prolonged survival of acutely SIV-infected animals was observed following IL-12 administration [164]. Expectedly, IL-12 therapy strongly influenced NK cell responses. Treatment with IL-12 during acute SIV infection augmented cytotoxic responses and increased numbers of NK cells. However, only partial restoration of NK cell functions was detected after IL-12 therapy during the late phase of infection due to the loss of cytokine responsiveness [165, 166].

Alternatively, combination therapy with IL-2 and IL-12 increased the capacity of activated NK cells to eliminate tumor cells [167]. IL-12 in combination with the proinflammatory cytokine IL-18 synergistically enhanced NK cell activation, IFN-γ production and proliferation, whereas during MCMV infection, activation of NK cells was more IL-18 than IL-12 dependent [168, 169].

IL-18 belongs to the IL-1 cytokine superfamily and is released early in response to viral infections [170]. It is constitutively produced in an inactive form (pro IL-18) and requires processing by the intracellular cysteine protease caspase-1 for maturation from the precursor into a biologically active molecule [171, 172]. The proinflammatory potential of IL-18 is constitutively antagonized by sustained secretion of the IL-18 binding protein (IL-18BP) [173].

Infection of macaques with simian/human immunodeficiency viruses resulted in a transient increase in IL-18 serum levels at primary viremia and elevated IL-18 production was associated with seroconversion [174, 175]. More precisely, the infection with HIV resulted in decreased IL-18BP concentrations and increased levels of biological active IL-18 [176]. It was demonstrated that IL-18 inhibited the production of HIV-1 p24 antigen in vitro [177] whereas during the chronic stage of HIV-1 infection, IL-18 directly stimulated viral replication [178]. In naïve mice increased FasL-mediated cytotoxicity of NK cells and upregulation of perforin-mediated NK cell activity was detected following IL-18 administration [179, 180]. In contrast, augmented IL-18 concentrations in chronically HIV-infected individuals were associated with increased death of NK cells [181]. Wang et al. [182] demonstrated that pre-treatment with IL-18 prior to HIV-1 infection could abrogate viral replication in vitro, predicting a potential for IL-18 treatment in HIV infection. Unfortunately, they did not analyze the involvement of NK cells in this study.

As described above, there is conflicting data about the effect of IL-18 monotherapy on NK cell responses. Nevertheless, NK cells treated with IL-18 in combination with IL-12 or triple therapy with IL-12, IL-15 and IL-18 resulted in strongly augmented NK cell degranulation and proliferation in cancer studies [183, 184]. It was also reported that pretreatment of isolated NK cells with IL-12, IL-15 and IL-18 resulted in increased antitumor activity, reduced tumor growth as well as cytokine-mediated induction of memory NK cells [185]. The efficiency and tolerability of cytokine administration depend on several factors such as administration routes, schedule of injections and the dosage. Administration of IL-12 could lead to flu-like symptoms, toxic effects on the liver and bone marrow in cancer patients, which are associated with the release of IFN-γ, TNF-α and chemokines [186]. Treatment of cancer patients with recombinant IL-15 may result in a reversible neutropenia but also in enhanced numbers of circulating NK cells and memory CD8^+^ T cells with minimal increases in Treg frequencies [187, 188]. In melanoma patients, IL-18 therapy in biologically active doses resulted merely in mild side effects, however, monotherapy showed only limited efficacy [189, 190]. Therefore, a cytokine combination therapy may show promising therapeutic effects but was not tested as anti-retroviral treatment until now.

## Conclusion

NK cells are important cytotoxic immune cells involved in the control of retroviral infections. Unfortunately, viruses developed numerous strategies to evade the immune pressure by cytolytic lymphocytes. Therefore, potent antiviral effects of NK cells in retroviral infections seem to be rather limited. Recently, therapeutic strategies to reactivate and improve NK cell functions were developed, mainly in cancer models. These new strategies are discussed in this review. Some of these approaches showed promising results in the first studies with retroviruses. A combination of these new therapies with ART might be an interesting future concept for achieving functional cure in patients that ultimately stop ART treatment.
